# Supplementary material for: Real-Time Fluorescence Microscopy on Living E. coli Sheds New Light on the Antibacterial Effects of the King Penguin β-Defensin AvBD103b
Source: Int J Mol Sci. 2022 Feb 12;23(4):2057. doi: 10.3390/ijms23042057 (PMC8880245; doi:10.3390/ijms23042057)
Supplement: Supplementary file 1 [file ijms-23-02057-s001.zip › ijms-1526496-supplementary.pdf]

Supplementary data

Real-time fluorescence microscopy on living *E. coli* sheds new light on the antibacterial effects of the King penguin β-defensin AvBD103b

Céline Landon[1,2*]; Yanyu Zhu[1,3], Mainak Mustafi[1,4]; Jean-Baptiste Madinier[2], Dominique Lelièvre[2], Vincent Aucagne[2], Agnes F. Delmas[2] and James C. Weisshaar[1]
1 Department of Chemistry, University of Wisconsin-Madison, Madison, WI 53706, USA
2 Center for Molecular Biophysics, CNRS, 45071 Orléans, France
3 Present address: Department of Bioengineering, Stanford University, Stanford, California, USA
4 Present address: Department of Biochemistry and Molecular Biophysics, Columbia University, New York, USA

Supporting Information Contents:

SUPP DATA 1: Mechanisms of action of β-defensins, a comprehensive survey of the literature (p2-5)

SUPP DATA 2: Antibacterial mechanism of AvBD103b does not involve significant stereo-selective interactions with any chiral partner (p6-7)

SUPP DATA 3: Example of slowing of GFP loss after *t1 (*p7)

SUPP DATA 4: Peptide Synthesis and Characterization of L- and D-AvBD103b (p8-11)
- General information
- General protocols for solid phase peptide synthesis
- Synthesis and characterization of AvBD103b and D-AvBD103b

REFERENCES: (p12)

Supplementary DATA S1: Mechanisms of action of β-defensins, a comprehensive survey of the literature

Only a few studies have attempted to dissect the mechanism of action of avian β-defensins, none performed on live targets and in real time (TABLE S1). In 2007, Van Dijk and co-workers [1] described for the first time the effect of an avian β-defensin (Chicken AvBD6, originally named Gallinacin-6) on the ultrastructure of the Gram+ bacteria *Clostridium perfringens.* The morphological changes observed by transmission electron microscopy (TEM) involve granulation of intracellular material, cytoplasm shrinkage, membrane leakage, irregular septum formation, and lysis at the septa of dividing cells. The absence of massive, complete lysis even at 4x MIC (Minimal Inhibitory Concentration), and the killing kinetics suggested that cell lysis itself is not the primary mechanism in AvBD6 mediated bacterial killing. Fluorescence-based biochemical assays on Gram- *E. coli* showed that the ostrich β-defensins (Ostricacin-1 and Ostricacin-2) at the MIC value permeabilize the *E. coli* outer membrane (OM) and induce a slow and partial depolarization of the cytoplasmic membrane (CM) [2]. This transient disruption of the CM might enable the peptides to access the cytoplasm and interact with intracellular targets such as DNA. More recently, morphological changes followed under scanning electron microscopy (SEM) indicate that the mechanism of killing of the Gram-bacterial strain *Aeromonas veronii* by the duck-billed platypus AvBD6 involves both cell lysis and non-lysis [3]. Finally, various ultrastructural changes were observed by TEM after the treatment of the Gram- bacteria *Salmonella typhimurium* with chicken AvBD6 and AvBD12. Symptoms included fuzzy membrane, vacuole formation, loss of cytoplasmic content and membrane blebbing/shrinking. The authors suggested that AvBDs not only disrupt bacterial membranes, but also interfere with intracellular functions, such as cell division [4].

A few additional studies conducted on other vertebrate β-defensins (TABLE S2), mainly human defensins, came to the same conclusion: compromise of the membrane initiates an irreversible killing process, but is probably not the primary cause of death [5-10].

Table S1: Rare studies reported in the literature to dissect the mechanism of action of avian β-defensins, done on various bacteria (either G+ or G-) and under various protocols and technics. TEM = Transmission electron microscopy, SEM = Scanning electron microscopy.

Avian β-defensin (origin) ***MIC***	***Bacteria***	***Methods*** **Peptide concentration** *Experimental conditions (Buffer/pH/Salts)*	***Main conclusions on the mechanism of action***	***Ref.***
**Gallinacin-6** *(Chicken)* MIC 8 µg/ml (1.9 µM)	*C. perfringens* *(G+)*	**TEM** **0 to 25 µg/ml of peptide** *30 min of incubation at 37°C in Trypticase Soy Broth (TSB) medium (pH not given), bacterial pellets prefixed in Karnovsky's reagent, stained, and embedded in durcupan resin.*	- Cell lysis itself is not the primary mode of β-defensin-mediated bacterial killing.	[1]
**Ostricacins-1 and 2** *(Ostrich)* MIC Osp1 0.3 µM MIC Osp2 0.2 µM	*E. coli* *(G-)*	**Fluorescence-based biochemical assays** **1 µM of peptide** *5mM HEPES, pH 7.2*	- Capable of permeabilize the outer membrane - Induce a slow and partial depolarization of the cytoplasmic membrane - not able to disrupt the bacterial membrane fully even at concentration higher than the MIC - The target could be the DNA	[2]
**GST-AvBD6** (*Anas platyrhynchos*) MIC 1 µM	*A. Veronii* *(G-)*	**SEM** **5x MIC of GST tagged peptide** *1 h of incubation at 37°C in TSB (pH not given); bacterial suspension fixed (0.1 M sodium cacodylate trihydrate buffer; pH 6.8), and dehydrated in ethanol.*	- The mechanism in GST-AvBD6 that mediated the killing of bacteria included both cell lysis and non-lysis.	[3]
**AvBD103b** *(King Penguin)* MIC 1.5 µM	*S. enteritidis* *(G-)*	**Outer membrane permeability assay** **2× or 4× MIC of peptide** *5 mM HEPES buffer pH 7.2 containing 5 mM glucose* **Flow cytometry** **1× to 5x MIC of peptide** *5 to 120 min of incubation, at 37 °C, in 10 mM PBS buffer (pH 7.2)* **TEM** **1×MIC of peptide** *2 h of incubation, at 37 °C in 100 mM PBS buffer (pH 7.2) before bacterial cells were stained, fixed and dried.*	- AvBD103b exerts its antibacterial activity by damaging the cell membrane and interfering with intracellular DNA, ultimately causing cell death. - Whether interference in cell-wall synthesis or interactions with other intracellular materials such as RNA or proteins are involved in the antibacterial mechanism of AvBD103b merits further investigation.	[11]
**AvBD6** *(Chicken)* MIC 16 µg/mL (3.4 µM) **AVBD12** (Chicken) MIC 128 µg/mL (26.1 µM)	*S. Typhimurium* *(G-)*	**TEM & SEM** **1×MIC of peptide** *30 min of incubation, at 37 °C, with 5mM NaCl (buffer and pH not given); bacteria fixed in Karnovsky's reagent, dehydrated with ethanol and stained*	- Membrane damage and cell deformation - Disrupted bacterial membrane but also interference with cell division and other intracellular functions that cause morphological changes	[4, 12]

Table S2: Main studies reported in the literature to dissect the mechanism of action of other vertebrate β-defensins, done on various bacteria (either G+ or G-) and under various protocols and technics. MIC = Minimal inhibitory concentration, MBC =* Minimum Bactericidal Concentration, *LC = Lethal concentration, TEM = Transmission electron microscopy, SEM = Scanning electron microscopy, nd = not defined

β-defensin (origin) & MIC	***Bacteria***	***Methods,*** **Peptide concentration** *Experimental conditions (Buffer/pH/Salts)*	***Main conclusions on the mechanism of action***	***Ref.***
**HBD1** (Human)<br>MIC *nd*<br>**HBD2** (Human)<br>MIC *nd*	*H. influenzae* (G-)<br>*M. Catarrhalis* (G-)<br>*S. pneumonia* (G+)	**TEM**<br>**10 µg/ml of peptide** (2.5µM for HBD1 and 2.3 µM for HBD1)<br>*30 minutes (HBD2) or 3 hours (HBD1) of incubation at 37°C, in Glutaraldehyde buffer 5% (pH 7.4); Bacterial pellets were fixed, dehydrated and embedded in Eponate*	- Innate immune molecules such as HBD1 and HBD2 can reduce the viability of major OM pathogens, perhaps by disrupting their membrane integrity.	[5]
**HBD2** (Human)<br>MIC 4 µM<br>**mfaBD2** (Macaque)<br>MIC 1 µM<br>**HBD3** (Human)<br>MIC 4 µM<br>**hcBD3** (Gibbon)<br>MIC 1 µM	*E. coli* (G-)	**Solute efflux kinetics & membrane-depolarization assays**<br>**5xMIC of peptide**<br>*10 mM sodium phosphate buffer, pH 7.0*	- Membrane poration capacity for the BDs in general, although they displayed different permeabilization kinetics.<br>- HDPs may produce, through interaction with membranes and a number of other low-affinity targets, inhibitory effects, which may result in additive or synergistic combinations during the killing process.	[8]
**HBD2** (Human)<br>MIC *nd*<br>**HBD3** (Human)<br>MIC *nd*	*E. coli* (G-)	**Flow cytometry**<br>**1 µM of peptide**<br>*5 mM sodium Phosphate buffer pH 7*	- Rapid compromising of the membrane, which is favored by the higher cationicity of hBD3, and which initiates an irreversible killing process, but which may not be the primary killing effect. A subsequent slower alteration of the bacteria may result in a later, more massive damage to the membrane.<br>- Interaction of the peptides with the membrane then leads to its permeabilisation, which may proceed in stages and only be the initial phase of the killing mechanism.	[7]
**HBD3** (Human)<br>MIC *nd*	*S. aureus* (G+)	**TEM**<br>**15 µM of peptide**<br>*10 to 60 min of incubation, at 37°C, in 10 mM sodium phosphate buffer (pH 7.4) with 20% Mueller-Hinton broth; bacterial suspension fixed (0.2 M sodium cacodylate trihydrate*	- Bacterial cell walls are synthesized by highly organized multienzyme machineries. hBD3 would interfere, like “sand in a gearbox,” with the coordinated assembly and function of the machinery, resulting in localized inhibition of cell wall biosynthesis and subsequent lesions.	[10]

		buffer; pH 7.4), and dehydrated in ethanol, and embedded in Araldite-Epon resin.		
**HBD4** (Human)<br>LC 5 µM	*E. coli*<br>*(G-)*<br>*P. aeruginosa*<br>*(G-)*	**Confocal microscopy**<br>**5 µM of peptide**<br>*10mM Phosphate buffer pH 7.4*<br>*images recorded after 10 min of incubation*	- Rapid localization of the labelled peptides inside the bacteria<br>- Transient interaction with microbial membranes.<br>- Membrane destabilization resulting in the formation of transient defects, which provide a pathway for rapid accumulation of peptides in the cytoplasm.<br>- Excess of cationic peptides in cytoplasm could then have an inhibitory effect on several metabolic processes leading to cell death.	[9]
**HBD1** (Human)<br>MBC 8 µM<br>**HBD2** (Human)<br>MBC 8 µM<br>**HBD3** (Human)<br>MBC 2 µM<br>**HBD4** (Human)<br>MBC 5 µM	*E. coli*<br>*(G-)*	**Time-lapse fluorescence confocal microscopy**<br>**Sub lethal concentrations : HBD1: 3 µM, HBD2: 3 µM,HBD3: 1 µM, HBD4: 3 µM;**<br>**And 2 × MBC of peptide**<br>*10 mM sodium Phosphate buffer pH 7.4*<br>**TEM**<br>**20 µM of peptides**<br>*10 mM sodium Phosphate buffer pH 7.4*	- Not all human β defensins permeabilize the *E. coli* inner membrane to the same extent (more extensive damage caused by HBD4 as compared to other defensins; HBD1 do not cause extensive damage).<br>- Mammalian defensins do not kill *E. coli* by a simple mechanism involving membrane permeabilization though their antibacterial potencies are very similar.	[6]

Supplementary DATA S2: Antibacterial mechanism of AvBD103b does not involve significant stereo-selective interactions with any chiral partner

At t = 1-3 min after starting a flux of 8 µM of D-AvBD103b, 10-50% of bacteria form periplasmic bubbles. At 4 µM, the bubbles are formed more slowly, on average after 19 min (11 to 34 min). Our results suggest that, as observed for the L-enantiomer, part of the well-structured compact 4.5 kDa D-AvBD103b crosses OM and reaches the periplasmic space, thus provoking invaginations of the CM. During this phase (Phase 1), the integrity of the OM is not significantly disturbed, as attested by the fluorescence of GFP, which generally does not leak outside (Fig. S3A, green curve). Following the formation (or not) of "bubbles" during phase 1, an abrupt decrease of about 10% in GFP intensity occurs at an average time t_1 of 13 min (range 9-18 min) after the start of exposure to 8 µM of D-AvBD10b. Exactly as described for the L-AvBD103b, the typical double-peaked transverse intensity profile of periplasmic GFP (Fig. S3B, black line) is suddenly replaced by a single narrower peak (Fig. S3B, blue line). GFP continues to leak during phase 2 (Fig. S3A, green curve). As for the L-isomer, the entry of Sytox (Fig. S3A, orange curves) always correlates with the loss of GFP. About 80% of the bacteria are labelled with Sytox at the end of the 1 h acquisition, showing that the CM is permeable to Sytox. This is the only slight difference with L-AvBD103b, for which 100% of cells are labeled with Sytox even at 2 µM.

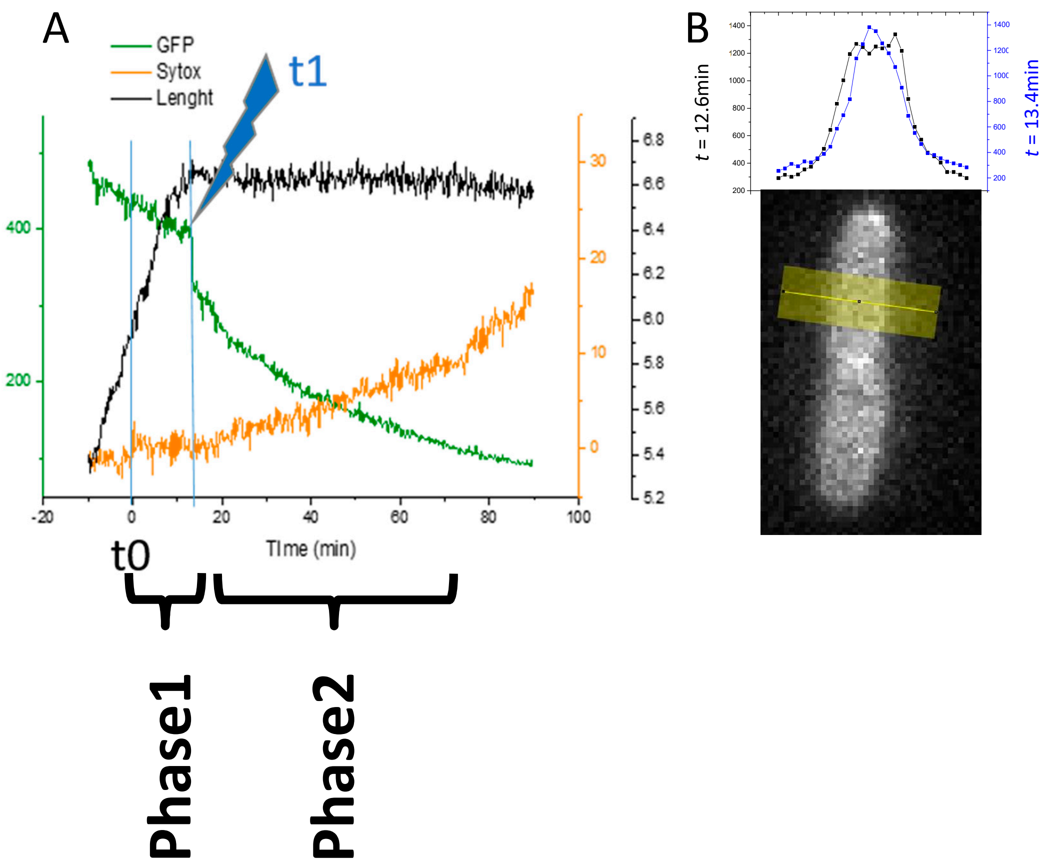

Supplementary figure S1: *Example of the effects of* D-*AvBD103b (at 8 µM) on representative* E. coli JCW10 *strain expressing periplamic GFP.* ***A.*** *Time dependence of total GFP intensity (in green, arbitrary units), of total Sytox intensity (in orange, arbitrary units) and of cell length (in black, in µm, calculated from phase contrast image). The flow of peptide begins at t = 0;* ***B.*** *For the same bacteria, transverse intensity linescans of GFP fluorescence (arbitrary units), following the yellow line, at time t = 12.6 min i.e. just before t1 (black line), and at time t = 13.4 min i.e. just after t1 (blue line).*

As for the L-isomer, explosions were observed for the D-isomer (about 10% of bacteria within the first hour), without any significant increase at the highest concentration tested. In two additional experiments, recorded at 4 µM, explosions occurred in the main field of view. In the first example, explosion occurs at t = 48 min, after the “t_1 event” (abrupt loss of GFP and beginning of slow increase of Sytox) which occurs at t = 40 min. In the second example, explosion occurs at t = 49 min, after the “t_1 event” recorded at t = 31 min (Supp data video).
In summary, the cascade of events is the same for D-AvBD103b as the L-isomer. Thus the antimicrobial mechanism of these peptides does not involve significant stereo-selective interaction with any chiral partner.

Supplementary DATA S3: Example of slowing of GFP loss after *t1*

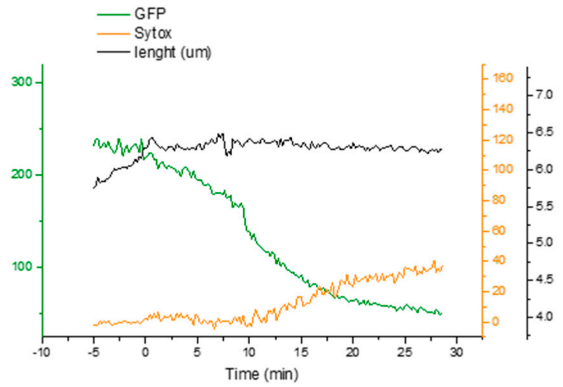

Supplementary figure S2: *Example of the effects of 1xMIC of* D-*AvBD103b on a representative* E.coli *JCW10 strain expressing periplamic GFP. The flow of peptide begins at t = 0. Time dependence of total GFP intensity (in green, arbitrary units), of total sytox intensity (in orange, arbitrary units) and of cell length (in black, in µm, calculated from phase contrast image).*

Supplementary DATA S4: Peptide Synthesis and Characterization of L- and D-AvBD103b

1) General information

All reagents and solvents were used without further purification. Protected amino acids, aminomethyl PEGA resin, HBTU and HATU were purchased from Merck Biosciences (Nottingham, UK). 3-(4-Hydroxymethylphenoxy)-propionic acid (MPPA) Wang-type linker was purchased from PolyPeptide laboratories (Strasbourg, France). Peptide synthesis-grade DMF, anhydrous DMF and anhydrous CH_2Cl_2 were purchased from VWR (Fontenay-sous-Bois, France). Ultrapure water was obtained using a Milli-Q water system from Millipore (Molsheim, France). All other chemicals were from Sigma Aldrich (St-Quentin-Fallavier, France) and solvents from SDS-Carlo Erba (Val de Reuil, France), and were used without further purification. Polypropylene syringes fitted with polypropylene frits were obtained from Torviq (Niles, MI, USA) and were equipped with PTFE stopcocks bought from Biotage (Uppsala, Sweden).

HPLC analyses were carried out on a Chromaster system equipped with a 5160 pump, a 5430 diode array detector and a 5260 auto sampler. Semi-preparative purifications were carried out on a LaChromElite system equipped with a Hitachi L-2130 pump, a Hitachi L-2455 diode array detector and a Hitachi L-2200 auto sampler. A Chromolith High Resolution RP-18e (150 Å, 10 × 4.6 mm, 3 mL/min flow rate) column was used for analysis and a Nucleosil C18 (300 Å, 5 µm, 250 × 10 mm, 3 mL/min flow rate) for purification. As mobile phase, mixtures of 0.1% TFA in H_2O (A) and 0.1% TFA in MeCN (B) were used.

High resolution ESI-MS analyses were performed on a maXis ultra-high-resolution Q-TOF mass spectrometer (Bruker Daltonics, Bremen, Germany), using the positive mode. The multiply-charged envelope was deconvoluted using the Charge Deconvolution algorithm in Bruker Data Analysis 4.1 software to obtain the monoisotopic [M] value.

Quantities of purified peptides were determined by UV spectrophotometry at λ = 280 nm (ε_{Trp} = 5500 and $\varepsilon_{SS\ bond}$ = 125 $L.M^{-1}.cm^{-1}$)[13].

Deoxygenation of solutions used for oxidative folding was performed through four consecutive vacuum (~5 mbar) / Argon cycles.

2) General protocols for solid phase peptide synthesis

- Introduction of the C-terminal Trp residue

In a syringe fitted with a frit, 3-(4-hydroxymethylphenoxy)-propionic acid (MPPA linker, 294 mg, 1.5 mmol, 5 equiv.) was coupled to aminomethyl PEGA resin (0.3 mmol, 1 equiv.) in the presence of HATU (570 mg, 1.5 mmol, 5 equiv.) and *i*Pr_2NEt (523 µl, 3 mmol, 10 equiv.) in 20 mL NMP. The syringe was stirred by rotation for 3 h, and the resin was then extensively washed with NMP. Completion of the reaction was checked by performing a Kaiser test on a few beads of resin [14]. Esterification of the Wang-type linker was then performed using the

symmetrical anhydride methodology, using anhydrous solvents and under an argon atmosphere: the MPPA-PEGA resin (0.15 mmol, 1 equiv.) was washed with DMF (3 x 10 mL), then 7 mL DMF was added and the resin kept solvated in the syringe. In a round bottom flask, Fmoc-Trp(Boc)-OH or Fmoc-*D*-Trp(Boc)-OH (1.58 g, 3 mmol, 20 equiv.) was dissolved in 10 mL of a CH_2Cl_2/DMF 9:1 mixture, and the resulting solution was cooled in an ice bath. DIC (189 µL, 1.5 mmol, 10 equiv.) was added and the reaction was stirred for 1 h at 0 °C. This solution was transferred by suction to the mppa-PEGA resin in DMF, followed by DMAP (1.8 mg, 0.015 mmol, 0.1 equiv.) dissolved in 1 mL DMF, and the syringe was stirred by rotation for 4 h. After extensive washing with DMF, capping of possibly unreacted hydroxyl groups was achieved through a treatment with acetic anhydride (429 µL, 4.5 mmol, 30 equiv.) in 10 mL of a DMF/pyridine 9:1 mixture for 2 h, followed by extensive washing with DMF then CH_2Cl_2. Fmoc group was removed by three successive treatments with 20% piperidine in NMP (12 mL) for 3 min. Effluents were collected and the fluorenylmethyl-piperidine adduct was quantified by UV spectroscopy at λ = 301 nm (ε = 7800 mol^{-1} cm^{-1}) in order to determine the esterification yield (0.128 mmol, 85 % in both cases, L- or D-tryptophan).

- Peptide elongation

Automated Fmoc-based solid phase peptide syntheses (SPPS) were carried out at a 0.1 mmol scale on a 431A synthesizer from Applied Biosystems. The following side-chain protecting groups were used: Arg(Pbf), D-Arg(Pbf), Cys(Acm), D-Cys(Acm), Gln(Trt), D-Gln(Trt), Ser(*t*Bu) and D-Ser(*t*Bu). Protected amino acids (1 mmol, 10 equiv.) were coupled using HBTU (360 mg, 0.95 mmol, 9.5 equiv.), HOBt hydrate (145 mg, 0.95 mmol, 9.5 equiv.) and *i*Pr_2NEt (348 µL, 2 mmol, 20 equiv.) in NMP (12 mL) for 30 min. Capping of possible unreacted amine groups was achieved by treatment with acetic anhydride (567 µL, 6 mmol, 60 equiv.), *i*Pr_2NEt (270 µL, 1.55 mmol, 15.5 equiv.) and HOBt hydrate (28 mg, 0.18 mmol, 1.8 equiv.) in NMP (12 mL) for 7 min. Fmoc group was removed by three successive treatments with 20% piperidine in NMP (12 mL) for 3 min.

- Solid-phase Acm deprotection

In a syringe fitted with a frit, the *S*-Acm-protected peptidyl resin (15 µmol) was swollen in NMP (2 x 5 mL for 1 min). Silver tetrafluoroborate (350 mg, 18 mmol, 120 equiv.) in an NMP/H_2O 9:1 mixture (4 ml) was transferred to the resin by suction, and the resulting slurry was stirred in the dark by rotation for 5 min at RT, followed by washes with NMP/H_2O 9:1. This treatment was repeated once (60 min stirring), and the resin was further washed with pyridine (5 x 6 ml), then treated alternatively with sodium diethyldithiocarbamate (25 mM in NMP) and pyridine hydrochloride (1 M in CH_2Cl_2/MeOH 95:5) (3 x 2 x 5 ml), followed by extensive washes with DMF.

- Peptidyl resin cleavage

Deprotection of side chains and cleavage from the resin was performed through a treatment with TFA/H_2O/*i*Pr_3SiH/phenol (88:5:2:5) for 2 h. The resin was filtered and the peptide was precipitated by dilution into an ice-cold diethyl ether, recovered by centrifugation, further washed three times with diethyl ether and dried under reduced pressure.

- Oxidative folding

The peptide (5 µmol) was incubated under argon with GSH (154 mg, 500 µmol, 100 equiv.) and GSSG (31 mg, 50 µmol, 10 equiv.) in 250 mL of a deoxygenated 100 mM Tris-HCl pH = 8.5

buffer containing 1 mM EDTA, for 20 h at room temperature. Reaction was quenched by addition of 1 mL TFA, then the folded peptide was purified by semi-preparative HPLC and lyophilized.

3) Synthesis and characterization of AvBD103b and D-AvBD103b

- AvBD103b

Sequence: H-[1]SFGLCRLRRGFCARGRCRFPSIPIGRCSRFVQCCRRV[38]W-OH

AvBD103b was synthesized following the general protocol, the coupling step being repeated once for Ser1, Arg9, Phe11, Cys12, Arg14, Arg16, Cys17, Phe19 and Ser21 during the automated Fmoc-SPPS elongation.

ESI-HRMS (*m/z*): [M] calcd. for $C_{194}H_{308}N_{70}O_{43}S_6$: 4498.2423, found: 4499.2398.
HPLC analysis: t_R = 3.56 min (Chromolith, gradient: 20-40% B over 5 min).
Overall yield: 10%

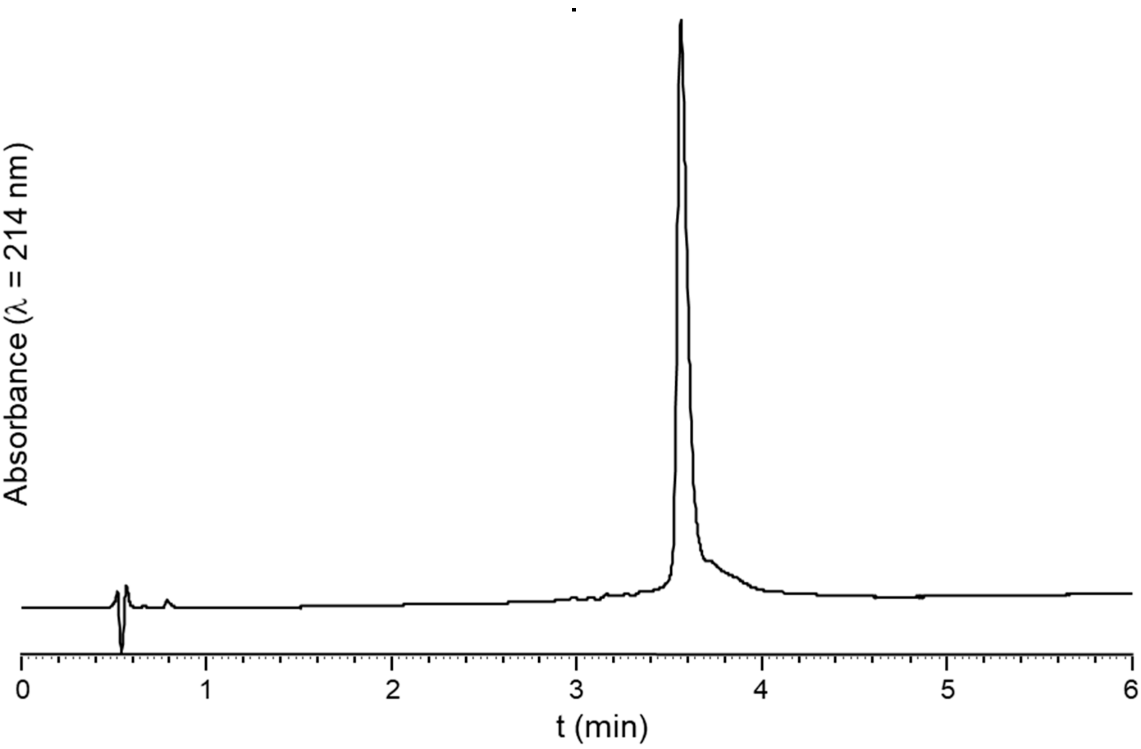

Supplementary figure S3: HPLC trace of purified AvBD103b.

- D-AvBD103b

Sequence: H-[1]sfGlcrlrrGfcarGrcrfpsipiGrcsrfvqccrv[38]w-OH

D-AvBD103b was synthesized following an identical protocol as for its *L*- counterpart, using D-amino acid building blocks.

ESI-HRMS (*m/z*): [M] calcd. for $C_{194}H_{308}N_{70}O_{43}S_6$: 4498.2423, found: 4499.2436.
HPLC analysis: t_R = 3.56 min (Chromolith, gradient: 20-40% B over 5 min).
Overall yield: 10%

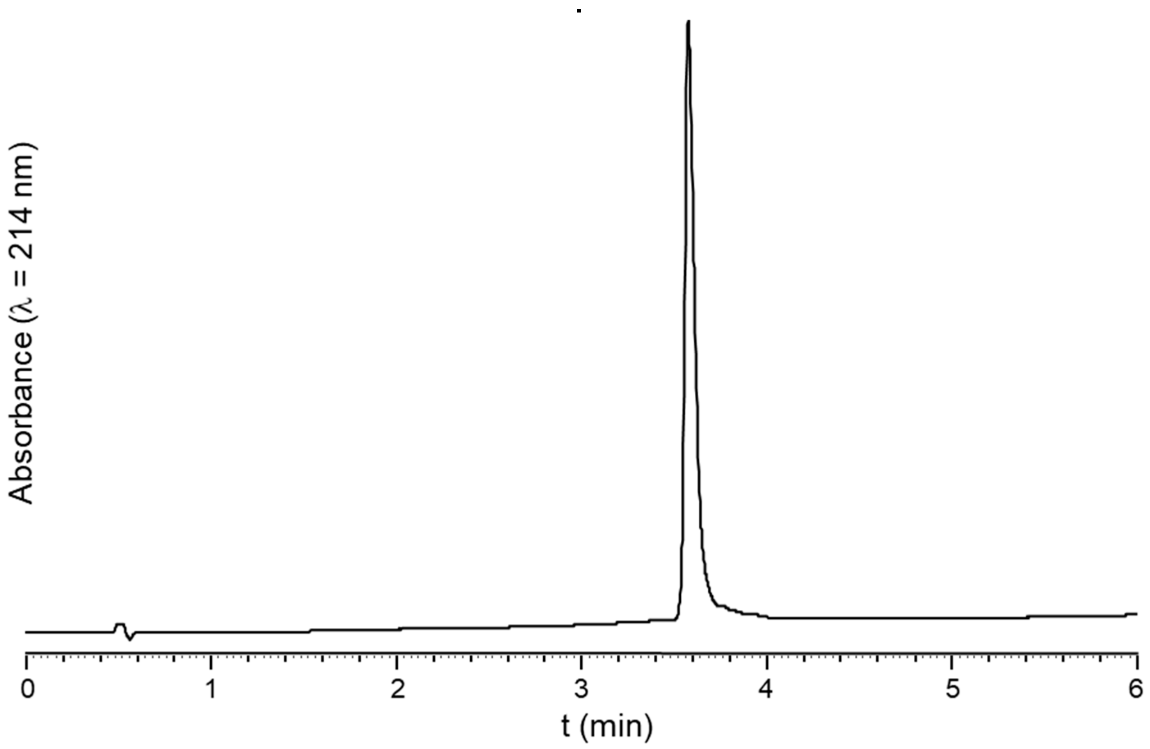

Supplementary figure S4: HPLC trace of purified D-AvBD103b.

REFERENCES:

1. van Dijk, A.; Veldhuizen, E. J.; Kalkhove, S. I.; Tjeerdsma-van Bokhoven, J. L.; Romijn, R. A.; Haagsman, H. P., The beta-defensin gallinacin-6 is expressed in the chicken digestive tract and has antimicrobial activity against food-borne pathogens. *Antimicrobial agents and chemotherapy* **2007,** 51, (3), 912-22.
2. Sugiarto, H.; Yu, P. L., Mechanisms of action of ostrich beta-defensins against Escherichia coli. *FEMS microbiology letters* **2007,** 270, (2), 195-200.
3. Peng, K. S.; Ruan, L. S.; Tu, J.; Qi, K. Z.; Jiang, L. H., Tissue distribution, expression, and antimicrobial activity of Anas platyrhynchos avian beta-defensin 6. *Poultry science* **2013,** 92, (1), 97-104.
4. Yang, M.; Zhang, C.; Zhang, X.; Zhang, M. Z.; Rottinghaus, G. E.; Zhang, S., Structure-function analysis of Avian beta-defensin-6 and beta-defensin-12: role of charge and disulfide bridges. *BMC microbiology* **2016,** 16, 210.
5. Lee, H. Y.; Andalibi, A.; Webster, P.; Moon, S. K.; Teufert, K.; Kang, S. H.; Li, J. D.; Nagura, M.; Ganz, T.; Lim, D. J., Antimicrobial activity of innate immune molecules against Streptococcus pneumoniae, Moraxella catarrhalis and nontypeable Haemophilus influenzae. *BMC infectious diseases* **2004,** 4, 12.
6. Mathew, B.; Nagaraj, R., Variations in the interaction of human defensins with Escherichia coli: Possible implications in bacterial killing. *PloS one* **2017,** 12, (4), e0175858.
7. Morgera, F.; Antcheva, N.; Pacor, S.; Quaroni, L.; Berti, F.; Vaccari, L.; Tossi, A., Structuring and interactions of human beta-defensins 2 and 3 with model membranes. *Journal of peptide science : an official publication of the European Peptide Society* **2008,** 14, (4), 518-23.
8. Sahl, H. G.; Pag, U.; Bonness, S.; Wagner, S.; Antcheva, N.; Tossi, A., Mammalian defensins: structures and mechanism of antibiotic activity. *Journal of leukocyte biology* **2005,** 77, (4), 466-75.
9. Sharma, H.; Nagaraj, R., Antimicrobial activity of human beta-defensin 4 analogs: insights into the role of disulfide linkages in modulating activity. *Peptides* **2012,** 38, (2), 255-65.
10. Sass, V.; Schneider, T.; Wilmes, M.; Korner, C.; Tossi, A.; Novikova, N.; Shamova, O.; Sahl, H. G., Human beta-defensin 3 inhibits cell wall biosynthesis in Staphylococci. *Infection and immunity* **2010,** 78, (6), 2793-800.
11. Teng, D.; Wang, X.; Xi, D.; Mao, R.; Zhang, Y.; Guan, Q.; Zhang, J.; Wang, J., A dual mechanism involved in membrane and nucleic acid disruption of AvBD103b, a new avian defensin from the king penguin, against Salmonella enteritidis CVCC3377. *Applied microbiology and biotechnology* **2014,** 98, (19), 8313-25.
12. Yang, M.; Zhang, C.; Zhang, M. Z.; Zhang, S., Novel synthetic analogues of avian beta-defensin-12: the role of charge, hydrophobicity, and disulfide bridges in biological functions. *BMC microbiology* **2017,** 17, (1), 43.
13. Pace, C. N.; Vajdos, F.; Fee, L.; Grimsley, G.; Gray, T., How to measure and predict the molar absorption coefficient of a protein. *Protein science : a publication of the Protein Society* **1995,** 4, (11), 2411-23.
14. Kaiser, E.; Colescott, R. L.; Bossinger, C. D.; Cook, P. I., Color test for detection of free terminal amino groups in the solid-phase synthesis of peptides. *Analytical biochemistry* **1970,** 34, (2), 595-8.
